# Supplementary material for: Cell-free RNA reveals host and microbial correlates of broadly neutralizing antibody development against HIV
Source: PLoS Pathog. 2026 Apr 9;22(4):e1014066. doi: 10.1371/journal.ppat.1014066 (PMC13065077; doi:10.1371/journal.ppat.1014066)
Supplement: Attachment — Submitted filename: Response_File.pdf [file ppat.1014066.s018.pdf]

Response to reviewers: PPATHOGENS-D-25-02483
Cell-free RNA reveals host and microbial correlates of broadly neutralizing antibody development against

Journal Requirements:

1) We ask that a manuscript source file is provided at Revision. Please upload your manuscript file as a .doc, .docx, .rtf or .tex. If you are providing a .tex file, please upload it under the item type u2018LaTeX Source Fileu2019 and leave your .pdf version as the item type u2018Manuscriptu2019.

2)Please upload all main figures as separate Figure files in .tif or .eps format. For more information about how to convert and format your figure files please see our guidelines: https://journals.plos.org/plospathogens/s/figures .

3) We have noticed that you have uploaded Supporting Information files, but you have not included a list of legends. Please add a full list of legends for your Supporting Information files after the references list.

1-3) We are now providing a .doc file as a manuscript source, and added eps figures. We have also included the legends of all supplementary figures after the reference list.

4) In the online submission form, you indicated that "The datasets generated and analyzed in the study are available in the NCBI Gene Expression Omnibus (GEO) and Sequence Read Archive (SRA) and can be accessed upon request". All PLOS journals now require all data underlying the findings described in their manuscript to be freely available to other researchers.

All data is now uploaded to GEO and we have generated a token for provisional access before publication. All code is also available from GitHub (https://github.com/CamunasLab/cfHIV)

To review GEO accession GSE313190:
Go to https://www.ncbi.nlm.nih.gov/geo/query/acc.cgi?acc=GSE313190
Enter token czineyqwrnelvob into the box

Major Issues:

Reviewer #1:

1. Success in cfRNA, cfDNA interpretation is control for RBC hemolysis and for release of platelet RNA. How were platelet and RBC contamination controlled for in this study? what percentate of RNA was from platelet or RBCs? In a paper by the senior author's group (PMID: 35132263) 60% of cfRNA was platelet + RBC derived.

To minimize confounding effects from hemolysis and platelet-derived RNA, we implemented several control steps. Plasma samples were visually inspected prior to processing for signs of hemolysis (e.g. pink discoloration), which was not visible in any sample. In addition, before cfRNA and cfDNA extraction, samples were submitted to a final centrifugation step (1 min at 16,000 rpm) to remove residual debris.

To further quantify the contribution of platelets and RBCs to the cfRNA signal, we performed a cell-type deconvolution analysis. We found that platelets and RBCs together account for 30-40% of the cfRNA signal in this dataset (Fig. R1), which is at the lower end of the range reported previously by our group for different cohorts (PMID 35132263; Extended Fig. 4, ~43%-70%, 3 cohorts).

Importantly, we did not observe significant differences in the platelet or RBC fractions between bNAb producers and non-producers (**Fig. R1**), indicating that group comparisons are unlikely to be confounded by differential hemolysis or platelet contamination. The same analysis revealed a modest increase in the neutrophil contribution in bNAb producers, compensated by decreased contributions from other cell types.

We believe this analysis strengthens the study and have incorporated it into the Results and Methods sections, with the corresponding data shown in Supplementary Figure S4. The analysis code is available in the GitHub repository (https://github.com/CamunasLab/cfHIV).

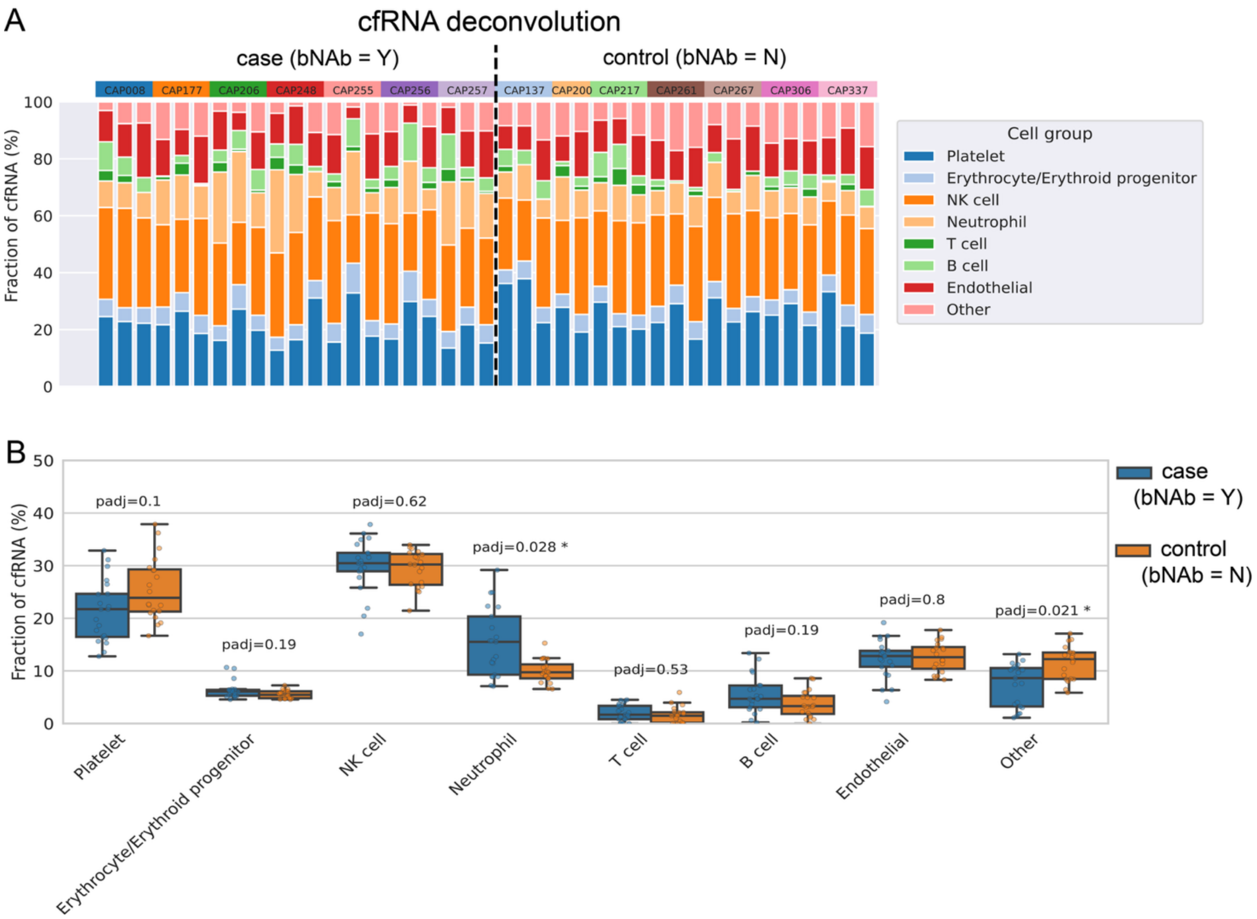

Figure R1. Cell-type deconvolution analysis of cfRNA. (a) Relative cell-type contributions to the cfRNA composition for each sample. Samples are grouped by participant and study group (bNAb producer or control), and less abundant cell types are aggregated into ‘Other’. (b) Box plots showing the relative abundance of each cell type in bNAb producers and controls. Statistical significance was assessed using a Mann–Whitney U test, with p-values adjusted for multiple hypothesis testing using Benjamini–Hochberg method.

Edits in Results, page 5 line 123:

‘Similarly, cell-type deconvolution of the host transcriptome showed comparable contributions of the most abundant cell types (e.g. red blood cells, immune cells, platelets) across both groups (Fig. S4).’

Edits in Methods, page 15 line 363:

“To minimize confounding effects from hemolysis and platelet-derived RNA, plasma samples were visually inspected prior to processing for signs of hemolysis (e.g. pink discoloration), which was not visible for any sample. Immediately before nucleic acid extraction, plasma samples were subjected to a final centrifugation step (1 min at 16,000 rpm) to remove any residual debris.”

2. That this study did not find an association between high viral load and CD4 counts with bnAb induction compared to other studies may only reflect the small sample size in this study. The authors did note that this was a pilot study and conclusions are limited.

We agree with the reviewer and have clarified this point in the Discussion. In page 14, line 324 we have now added:

"*For example, associations between viral load or CD4 counts and bNAb induction reported in other studies may not be detectable here due to the limited size of this pilot cohort.*"

3. In Figure 1A the difference in those with bnAbs and those with fewer bnAbs is not well demarcated. In 1A the lowest breadth % of the bnAb group and the highest breadth % of the low neutralizing group overlap at 3 years of follow up. That is unlike other such studies, as here, there is no clean break between the groups.

Participants were classified as bNAb producers if they achieved ≥20% breadth within year of infection or ≥40% breadth against the 18-pseudovirus panel at any three years post-infection.

4. In Figure 1B, it is unclear what the boxes mean, and what log 10 affinity means in the context of pseudovirus neutralization assays. Does affinity correlate with EC50 or EC80?

We thank the reviewer for pointing this out. In Figure 1B, each box represents the IC50 neutralization titer measured in a pseudovirus neutralization assay (rather than a binding affinity). As is standard for neutralization titers, a logarithmic scale is used for visualization given the broad dynamic range of the measurements, with higher log10(IC50) values indicating stronger neutralization potency. Samples are grouped by participant and study group to highlight the development of broadly neutralizing responses in bNAb producers (top half). We have now edited Figure 1 to correct the label and make the content of the caption clearer.

5. Figure 3A, it is unclear what test was used for the p values shown.

We now specify in the caption of Figure 3 that the correlation coefficients and p-values shown in all panels are based on Spearman's rank correlation analysis.

6. Figure 4, correlations are presented but no tests are indicated how they were obtained.

We have also clarified the caption of Figure 4 to indicate that correlations with breadth were assessed using Spearman's rank correlation analysis, and we now refer to the Methods section, where the statistical package used for the analysis (SciPy, Python) is specified.

7. In Figure 5 there does not appear to be statistically significant differences in GBV-C levels in the two groups. No statistical methods noted to analyze the data.

As noted by the reviewer, this result was not previously supported by a formal statistical analysis. We have now analyzed the data using a logistic mixed-effects model with participant as a random effect to account for repeated sampling. Using the classification shown in Figure 5, we found that bNAb producers had higher odds of increased GBV-C levels compared to non-bNAb producers (odds ratio [OR]: 6.4, 95% CI: 2.3-17.9). We have added this information into the Results section, figure caption and Methods section under "GBV-C analysis". Specifically, we have modified the Results section and caption in the following way:

Page 10 line 252:

In our study, all bNAb producers had non-zero levels of GBV-C ($p < 0.05$, one-sided Fisher exact test) and showed higher odds of elevated GBV-C levels (Figure 5B).

Caption Figure 5:

"Using a logistic mixed-effects model with participant as a random effect, bNAb producers showed higher odds of elevated GBV-C levels (odds ratio [OR]: 6.4, 95% CI: 2.3-17.9). In some participants, GBV-C read counts exceeded those of HIV up to 100-fold."

8. In Figure S2 there appear to be as many sequences in the water control as in the samples. Is this usual? How are relevant cf genetic sequences identified with such high background of contamination? 9. Also in Figure 2 why were so many contaminating sequences "cleaned" from the bnAb group but not in the control group?

To address concerns regarding potential differences in preprocessing QC steps and background contamination in cfRNA, we have performed a more in-depth analysis of complementary metrics.

First, the apparent similarity in total cfRNA read counts between plasma samples and negative controls (water samples) in Fig S2 arises from two factors: the use of a log scale (which visually compresses differences) and the fact that raw counts primarily reflect library input into the 'sequencing pool' (total volume or biomass used). We intentionally included negative controls with sufficient input and sequencing depth to capture a representative signature of potential background reads from reagents and environment. Even with this enrichment approach, negative controls have substantially fewer reads (**Fig. R2a,b**) and a lower fraction of human-mapped reads than plasma samples (**Fig R2c**). The number of human-mapped reads observed in negative controls (<0.5% of

total reads) is compatible with index-hopping in the Illumina sequencing platform rather than sample cross-contamination. More importantly, we see no differences in any of these parameters between bNAb producers and non-producers (**Fig. R2**) ruling out confounding effects.

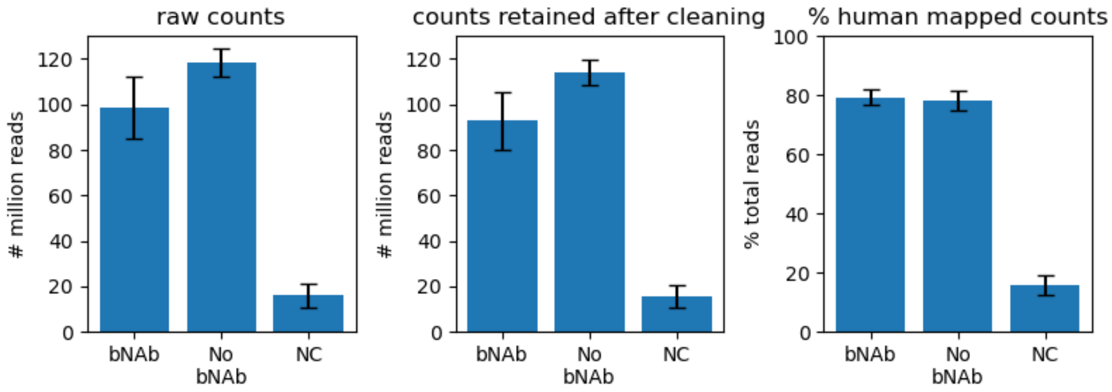

Figure R2. Sequencing depth and quality control metrics in cfRNA. Total raw reads counts **(left)**, reads retained after cleaning **(middle)**, and % of reads mapped to the human genome **(right)** for each study group. Negative controls (NC) show lower read counts and human-mapped fractions compared with plasma samples, while no systematic differences are observed between bNAb producers and non-producers (Mann-Whitney U test, p>0.05). Data are shown as mean ± SEM.

We also examined the sequencing quality control steps for both cfRNA and cfDNA. The cleaning step consists of standard library adapter trimming, read-pair merging, and removal of reads mapping to the UniVec Core database, and does not involve any taxonomic filtering. In cfRNA libraries, this step removes a small fraction of reads (<10%) in both plasma samples and negative controls, indicating that most reads pass basic quality control (**Fig. R3**). In 3 plasma samples, we found a higher abundance of adaptors (~25%, Fig. S2). These samples belong to different participants (both bNAb/non-producers), and likely reflect lower sample quality. In contrast, cfDNA water controls contain a larger fraction of library adapters that are removed in the 'cleaning' step. This explains the different QC patterns observed in negative controls between cfRNA and cfDNA.

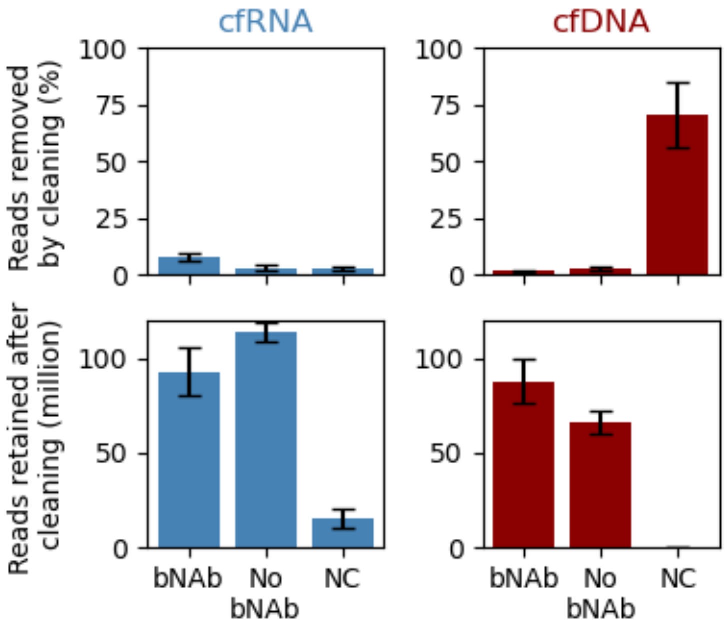

Figure R3. Quality control metrics for cfRNA and cfDNA libraries. (Top) Percentage of reads removed in the cleaning step for cfRNA (left) and cfDNA (right) libraries per group **(Bottom)** Number of reads retained after cleaning for cfRNA (left) and cfDNA (right) libraries. Data is mean ± SEM, and groups are bNAb producers (bNAb), non-producers (No bNAb) and negative controls (NC).

All subsequent taxonomic analyses focused on cfRNA data. To minimize potential confounding effects of background RNA, we performed a differential abundance analysis between negative controls and plasma samples. This analysis was characterized by taxa strongly enriched in the negative controls ($|log_2FC|>4$, $FDR<10^{-8}$), which we used to defined background contamination levels (**Fig. R4a**). Microbial reads observed in this background signal included environmental taxa commonly detected in DNA extraction kits and reagents in low-biomass sequencing (e.g. Robbsia, Burkholderia, Ralstonia) (Salter et al, BMC Biol, 2014).

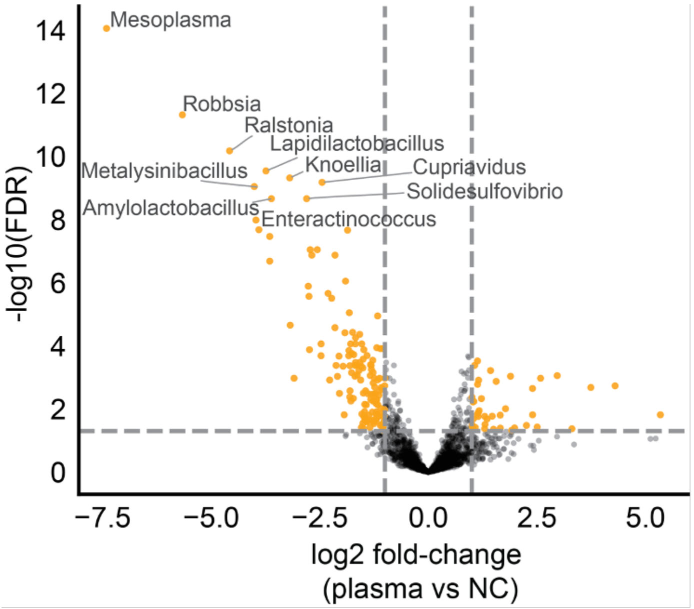

Figure R4: Background microbial sequences in negative controls. Volcano plot showing differential abundance of microbial genera between plasma samples and negative controls (NC) (FDR < 0.05, Benjamini–Hochberg). Negative log2 fold-change values indicate genera enriched in NC.

In contrast, differential analysis between bNAb producers and non-producers identified taxa associated with each group with more modest effect sizes (1<|logFC|<3, FDR< 0.05) and different from the background signature observed in negative controls (**Fig. R5a**). These included bacteria commonly found at mucosal surfaces and in the gastrointestinal tract, such as Lactobacillus and Enterobacteriaceae (**Fig. R5a,b**). Together, these analyses demonstrate that the bNAb-associated microbial signal is distinct from background contamination and is not driven by other technical aspects such as sequencing depth and differential preprocessing. Additional results specific to the viral component of the microbial analysis are further discussed in the answer 4 to reviewer 2.

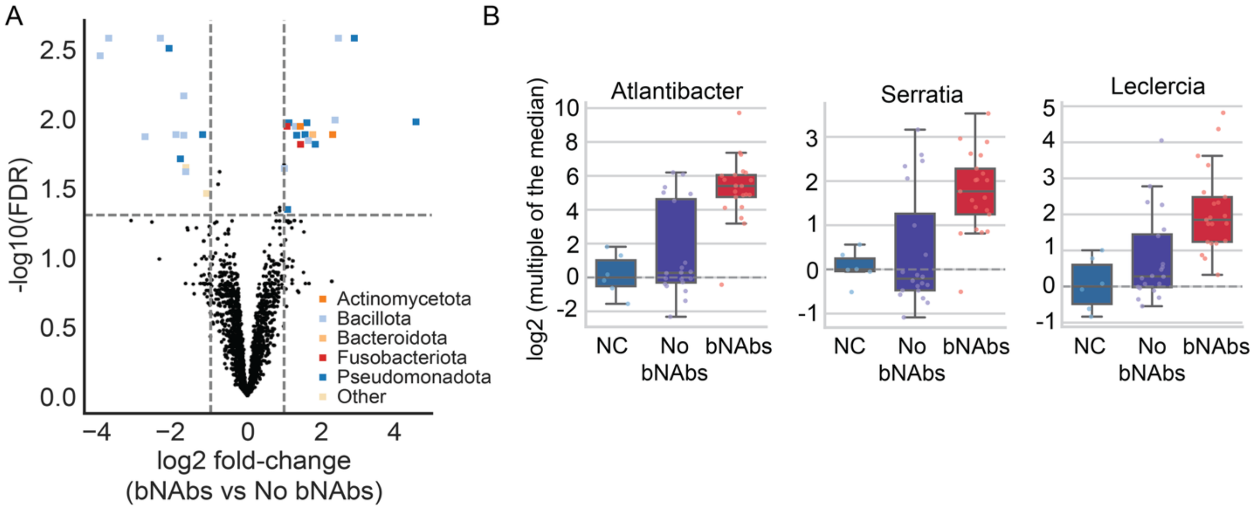

Figure R5: Differentially abundant microbial sequences in bNAb producers. A) Volcano plot of differentially abundant microbial genera between bNAb producers and non-producers, colored by phylum (FDR < 0.05,

Benjamini-Hochberg). Positive log2 fold change indicates enrichment in bNAb producers. B) Boxplots showing normalized abundances of selected microbial genera across negative controls (NC), bNAb non-producers, and bNAb producers. Values are shown as log2-transformed abundances relative to the median abundance in negative controls.

[REF1] Salter SJ, Cox MJ, Turek EM, Calus ST, Cookson WO, Moffatt MF, et al. Reagent and laboratory contamination can critically impact sequence-based microbiome analyses. BMC Biology. 2014;12:87

10. In Figure S3, was the RNA outside of cells and outside of virions or does this sequencing simply represent virion-associated RNA? Were the samples pelleted by ultracentrifugation to remove intact virions?

We did not pellet plasma samples by ultracentrifugation to remove intact virions; therefore, our workflow could capture virion-associated RNA, viral RNAs released into plasma from infected cells, and other extracellular RNAs. Additionally, our library preparation method is based on random priming without a fragmentation step and uses high-processivity enzymes and, in principle, could capture both long RNA molecules (potentially including near full-length viral fragments) and short RNA fragments.

However, paired-end reads mapping to HIV predominantly span short genomic regions (~300 bases, Fig. R6**a**) and we do not find systematic relationships between genomic span and start position that could indicate priming from long RNA templates (**Fig. 6b**). This argues against a library dominated by intact full-length virion-associated RNA, and is most consistent with priming from pre-fragmented RNA templates. In line with this, we do not observe increased read accumulation towards the 3’ end of the HIV genome, nor clear patterns indicative of abundant spliced viral transcripts (**Fig R6c**).

While we cannot exclude that a fraction of reads derives from virion-associated RNA molecules, the observed read length and coverage profiles are most consistent with predominantly fragmented cell-free viral RNA rather than intact virion genomes. We have now included this analysis in supplementary figure S6.

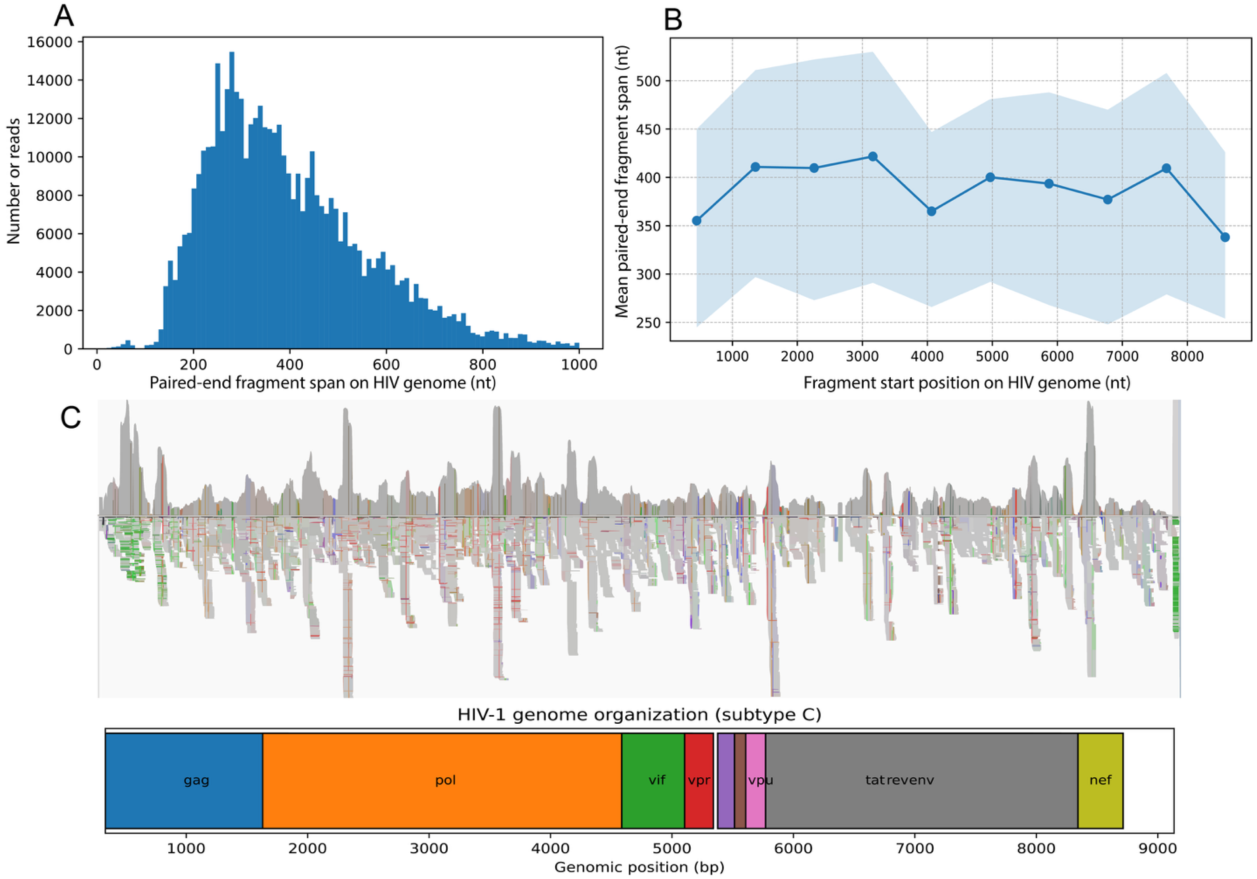

Figure R6. Fragment length and coverage of cfHIV reads. (a) Distribution of paired-end genomic spans for reads mapping to the HIV genome, showing a predominance of short fragments (200-600 bp). (b) Mean paired-end genomic span as a function of genomic start position, showing no systematic trends that could indicate priming from long RNA templates. (c) Genomic coverage profile across the HIV genome, shown alongside the reference organization of the HIV genome.

11. Figure S6 is hard to understand. Are these data from only the PLWH who made high bnAbs? Or both groups?

Former figure S6 (now S11) includes all reads mapping to non-human references pooled across both groups (PLWH who do and do not produce bNAbs) and is intended to illustrate the overall distribution of non-human mapping reads rather than group-specific differences. We have now clarified this in the figure caption. Following the analyses performed in response to Q8, we have added additional supplementary figures that complement this figure and help clarify findings from the microbial sequence analysis.

12. Figure S7. What are pseudo-temporal dynamics? Definition would help the reader.

Pseudotime analysis is a trajectory-inference approach in which samples are ordered by relative biological state rather than chronological time. In our study, samples were ordered based on plasma neutralization breadth (number of viral strains neutralized), which was used as a proxy for progression along the trajectory of bNAb development. This approach accounts for inter-individual differences in the pace of bNAb development, as identical sampling time points may correspond to different stages of bNAb development across individuals. We have clarified this concept in the Results, Methods, and figure caption. Specifically, we have included the following definition in the results section (line 195):

'To investigate this immune activation in more detail, we performed pseudotime analysis on the two groups, using plasma neutralization breadth to define the temporal progression (Figure 4B, Methods). Pseudotime is a trajectory-inference approach that orders samples by relative biological state rather than chronological time, allowing comparison across individuals at different stages of bNAb development. We found that the immunologic gene signature is elevated only early in breadth development in bNAb producers (when only a limited number of viral strains are neutralized) and later converges to levels comparable to non-bNAb producers as neutralization breadth increases.'

13. Several uses of the word "significantly" (as in line 193) with no reference to statistical test or giving a P value. Similarly the word "correlated" or "anti-correlated" was used (as in lines 209-210) with no correlation coefficient or statistical test described.

We have revised the Results text to explicitly state the statistical test and p-value thresholds used for correlation analyses in the corresponding figure legend (Spearman correlation with Benjamini–Hochberg). We have also added explicit references to p-value thresholds in sections where statistical results are described in the text without direct reference to a figure. Finally, we have removed uses of the term "significant" in cases where it was redundant or lacked explicit statistical support.

Reviewer #2: (Fabio Zanini)

1. Data should be on GEO with identifier, and code should be available e.g. on GitHub.

We have uploaded the data to GEO with identifier GSE313190 and it will be made publicly available upon publication. A token for reviewer access has been generated with the following token:

To review GEO accession GSE313190:
Go to https://www.ncbi.nlm.nih.gov/geo/query/acc.cgi?acc=GSE313190
Enter token czineyqwrnelvob into the box

We also have uploaded all code needed to reproduce the figures in a publicly available GitHub repository (https://github.com/CamunasLab/cfHIV). This information has been included in the data availability section of the paper.

2. Although the authors demonstrate that they can reconstruct HIV genomes using cell-free nucleic acids, it is unclear to me how many reads they need to achieve what kind of assembly quality. Some downsampling to get a sense of how many cfRNA reads you would need to assemble an HIV genome to what degree/quality would really help future researchers tailor their experimental design.

This is an aspect that we had not explored. We have now performed a subsampling analysis using the sample with the highest number of HIV-mapped reads and evaluated HIV genome coverage at multiple depth thresholds (Figure R7).

Specifically, we subsampled HIV-mapped reads down to 1% of sample CAP261 and assessed the fraction of the HIV genome covered at different depth thresholds (Figure R7A). This analysis shows that read counts corresponding to approximately 50,000 HIV-mapped reads are sufficient to achieve ~90% genome coverage at ≥20X–50X depth. As a quality check, we also verified that there is a linear relationship between the number of HIV-mapped reads and mean coverage depth across the HIV genome (Figure R7b).

Finally, we evaluated how these metrics translate to a real dataset by examining coverage across the full cohort. We found that 83% of samples achieved >90% HIV genome coverage at ≥10X and ≥20X depth, and 69% at ≥50X depth (Figure R7c). These coverage levels were obtained using sequencing depths typical of most cfRNA studies (~50–100 million paired-end reads per sample). We have included this analysis as a supplementary figure, as suggested by the reviewer, as we believe that it may be useful for other groups planning cfRNA-based viral sequencing studies.

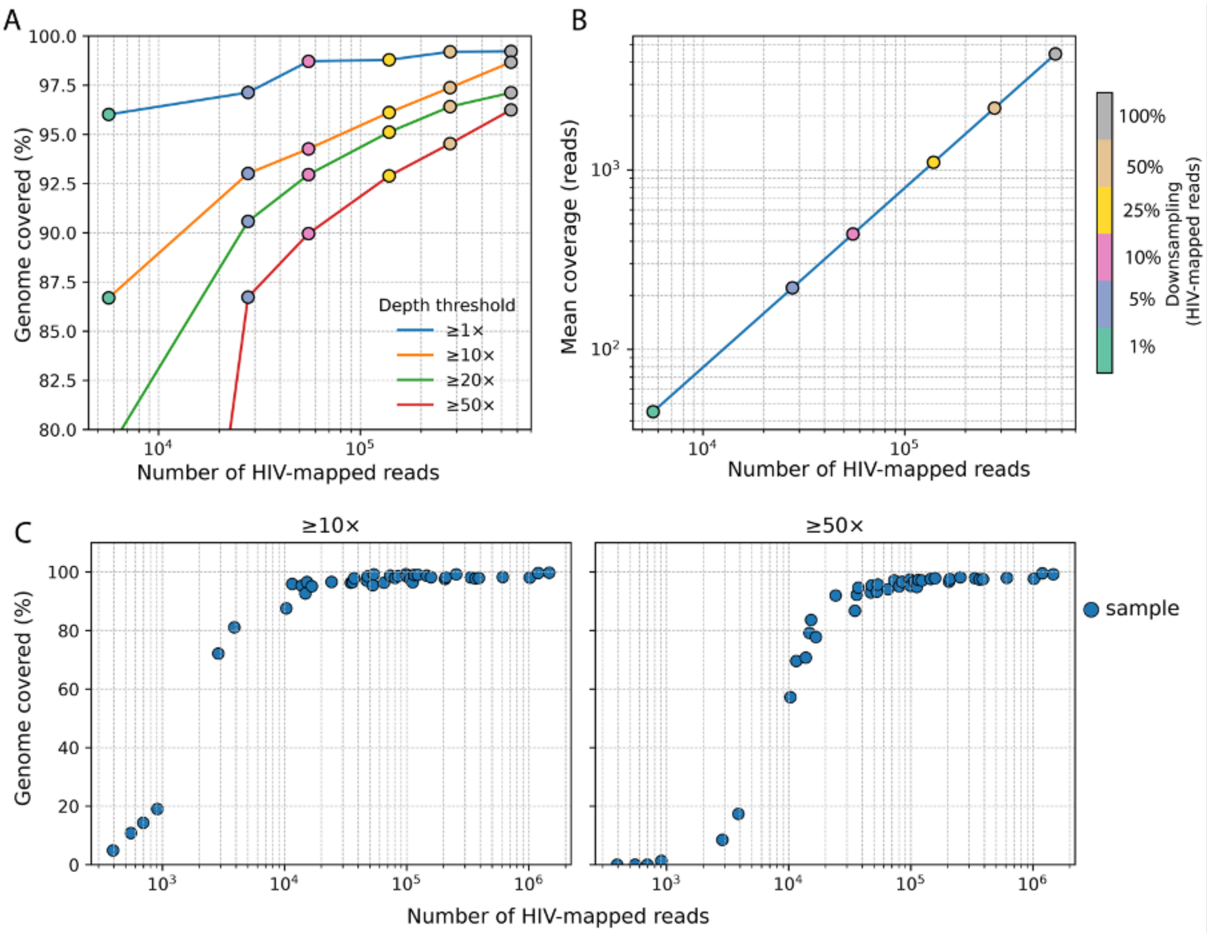

Figure R7. Subsampling analysis of HIV genome coverage from cfRNA. A) Fraction of the HIV genome covered above increasing minimum depth thresholds (1X-50X) at different subsampling levels of sample CAP261. B) Number of HIV-mapped reads versus mean coverage depth across the HIV genome, showing a linear relationship. C) Genome coverage achieved for each sample in the cohort at ≥10X (left) and ≥50X (right) depth thresholds.

3. On line 170, the authors state that CCL5 etc. are responses to HIV infection. Because these genes have very broad immune functions, the authors should explore the immunology literature to clarify whether these genes are responses not just shared between HIV bNAb groups, but also with patients infected by other viruses or even bacteria. The authors should comment on this issue based on a literature search and potentially cross-examine third-party data from previous publications to check the specificity of this signature.

We thank the reviewer for this important point. We agree that genes such as CCL5 have broad immune functions and are components of conserved host responses to viral infection, rather than being specific to HIV alone. We have revised the Results text accordingly to clarify that these genes are interpreted as shared host transcriptional responses associated with peripheral viral burden, rather than HIV-specific markers. We have edited the following sentences:

Caption Figure 3 B: *"Subset of genes from panel A previously described as involved in host immune responses to viral infection."*

Caption Figure 4B: "Labelled points are genes involved in host immune responses to viral infection."

Page 7 line 157: "Analysis of genes significantly correlated to cfHIV revealed 19 genes typically associated with host immune responses to viral infection, whereas only 1 for each of viral load and CD4 count, respectively (Figure 3B)."

Page 7 line 167: "These genes do not show a differential behaviour between participants who develop bNAbs and those who did not, indicating that they represent shared responses to HIV infection. This shows that cfRNA sequencing captures shared host transcriptional responses associated with peripheral viral burden during HIV infection."

Page 11 line 277: "We find that overall cfHIV levels—defined as HIV-derived fragments in cfRNA—correlate with abundance of host genes involved in immune responses to viral infections, including CCL5, TFRC, and CCL2, consistent with responses to peripheral viral burden."

4. The authors mention GBV-C but do not elaborate whether other flaviruses were also found. HCV-HIV coinfection is not that uncommon and would cause a quite different immunological response, therefore the authors should clarify whether they specifically see Hepatitis C reads in their samples.

We thank the reviewer for bringing up this point, especially given the clinical importance of HCV–HIV coinfection. We first reviewed available clinical metadata but HCV status was not captured in the database. However, we detected only very low numbers of reads mapping to HCV in cfRNA. Upon closer inspection, these reads were localized to specific, narrow regions of the HCV genome with low sequence complexity, and they most likely represent misalignments or technical artifacts rather than viral signal (**Figure R8a)**. In contrast, reads mapping to GBV-C showed broad coverage across the viral genome with consistent depth profiles across samples (**Fig. R8b)**, consistent with the presence of viral RNA. Altogether, these results indicate that the presence of GBV-C RNA and its associations with the host transcriptome are unlikely to be confounded by HCV–HIV coinfection.

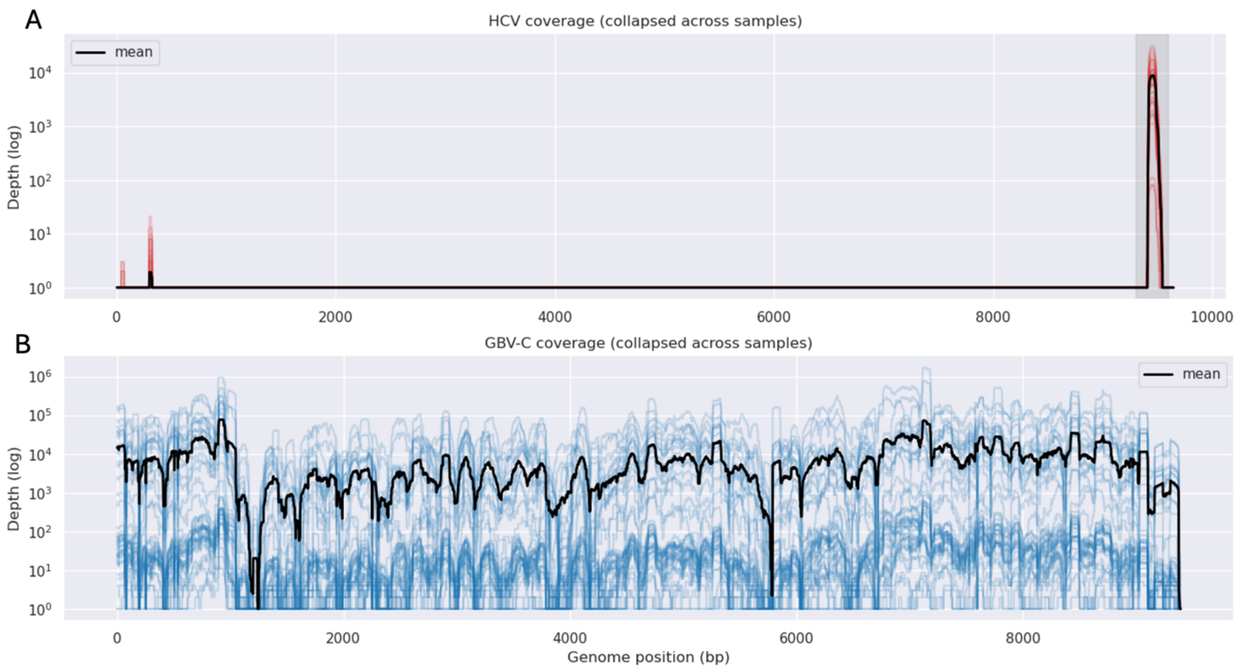

Figure R8. Coverage profiles of HCV and GBV-C genomes across samples. (a) Read coverage across the HCV genome (b) Read coverage across the GBV-C genome. Coverage for individual samples is shown in red (HCV) and blue (GBV-C); mean coverage is shown in black. HCV coverage is sparse and localized in low complexity regions of the genome, whereas GBV-C shows broad genome-wide coverage.

5. Did the pseudovirus neutralisation assay in Figure 1B for non-bNAb producers match the strain that was found in their cfRNA reads? In other words, can their serum at least neutralise narrowly their own strain?

We and other have previously shown that HIV infection, in these specific participants, and generally, results in neutralization of the autologous strain within weeks. This is true of bNAber and non-bNAber, and the titer of the autologous response does not differentiate these two groups (Moore et al, JV, 2008; Moore et al, PLOS Path, 2009, Moore et al, JV, 2011, as examples from this cohort).

Examples from this cohort:

[REF2] Moore et al. The c3-v4 region is a major target of autologous neutralizing antibodies in human immunodeficiency virus type 1 subtype C infection. J Virol. 2008 Feb;82(4):1860-9.
[REF3] Moore et al. Limited neutralizing antibody specificities drive neutralization escape in early HIV-1 subtype C infection. PLoS Pathog. 2009 Sep;5(9):e1000598.
[REF4] Moore et al. Potent and broad neutralization of HIV-1 subtype C by plasma antibodies targeting a quaternary epitope including residues in the V2 loop. J Virol. 2011 Apr;85(7):3128-41.

Earlier references:

[REF5] Wei et al. Antibody neutralization and escape by HIV-1. Nature. 2003 Mar 20;422(6929):307-12.
[REF6] Richman at al. Rapid evolution of the neutralizing antibody response to HIV type 1 infection. Proc Natl Acad Sci U S A. 2003 Apr 1;100(7):4144-9.

[REF7] Li et al. Evidence for potent autologous neutralizing antibody titers and compact envelopes in early infection with subtype C human immunodeficiency virus type 1. J Virol. 2006 Jun;80(11):5211-8.
[REF8] Gray et al. Neutralizing antibody responses in acute human immunodeficiency virus type 1 subtype C infection. J Virol. 2007 Jun;81(12):6187-96.

Minor Issues: Editorial and Data Presentation Modifications Please use this section for editorial suggestions as well as relatively minor modifications of existing data that would enhance clarity.

Reviewer #1:

15. The participants in the CAPRISA cohort study are referred to as either "donors" or "patients" in the paper (line 235). Perhaps the generic term "participants" might be more consistent.

We agree with the reviewer and we have homogeneized the text by using the form 'participants'.

16. Vague phrases throughout. Line 43, "activation that involves MHC class I antigen presentation"; line 62 "changes in commensal microbiota and increased GB virus C co-infection" (increased levels or increased number of participants?); GB virus C is mentioned several times in the paper before it is defined in line 251. It should be defined early on in the paper.

We thank the reviewer for noticing these ambiguities, and we have revised the abstract and author's summary to make the text more precise. The revised text now reads:

Abstract

Line 39: "*We find that development of bNAbs is associated with a transcriptomic signature of early immune activation characterized by elevated levels of MHC class I antigen presentation genes.*"

Line 44: "Finally, we also identified an enrichment of several microbial taxa in bNAb producers and increased levels of GB virus C (GBV-C), a non-pathogenic lymphotropic virus."

Author's summary:

Line 57: "Early in infection, bNAb producers showed an MHC class I-linked immune activation signature that declined over time, alongside changes in commensal microbial sequences and increased levels of GB virus C in cfRNA."

Reviewer #2:

The first sentence of the abstract is not strictly true: a narrow response is ok to prevent viral infections if the strain matches. Hence the yellow fever vaccine. Rephrasing is needed.

We agree that strain-specific antibody responses can be sufficient to prevent infection when well matched to the infecting virus. To avoid an overstatement, we have removed the first sentence of the abstract.

Not sure CCL5 etc. are "covariates of HIV infection progression" as much as they are proxies of high viral titers in the periphery. The two things are not quite the same as progression is clinically defined based on CD4 counts rather than viral load.

We agree with the reviewer and have revised the text accordingly to clarify that these genes reflect host immune responses to viral burden rather than markers of clinical disease progression. Specifically, the Results section now reads:

Page 7 line 157: "Analysis of genes significantly correlated to cfHIV revealed 19 genes typically associated with host immune responses to viral infection, whereas only 1 for each of viral load and CD4 count, respectively (Figure 3B)."

Page 7 line 167: "These genes do not show a differential behaviour between participants who develop bNAbs and those who did not, indicating that they represent shared responses to HIV infection. This shows that cfRNA sequencing captures shared host transcriptional responses associated with peripheral viral burden during HIV infection."

The authors dutifully cite many of the software packages used for the analyses but miss others. For instance, they presumably used HTSeq 2.0 but fail to cite it. They should amend this inconsistency.

We have revised the bibliography to include the missing citation to HTSeq and ensure consistent citation of all software tools.

The discussion section about lymph nodes is highly speculative and should be tempered to avoid giving the impression that this manuscript provides hard evidence that specific signature found herein can be attributed unequivocally to lymph node processes.

We have now tempered the discussion section to better reflect the provided level of evidence. Specifically we have edited the paragraph starting in page 12 line 313 that now reads:

"Previous studies have highlighted the central role of lymph nodes in shaping humoral responses during HIV infection, yet their direct study remains challenging (19,51,52). Cell-free RNA may provide a non-invasive window into immune activity occurring in tissue compartments, including lymphoid tissues where Tfh-B cell interactions drive affinity maturation. Hence, it is plausible that the immune-related cfRNA transcripts we report here reflects immune activity within lymph nodes, consistent with previous evidence that cfRNA can capture tissue-resident hematopoietic activity such as bone marrow progenitor transcripts (33). However, we emphasize that cfRNA represents an integrated signal from multiple sources, and we cannot exclude the possibility that increased cfRNA levels are due to contributions of circulating immune cells or other tissues."

In addition to the above, I have a specific suggestion that, albeit not 100% necessary for the manuscript, would elevate the conclusions of the paper and potentially shed light on new biology:

• The correlation between higher MHC-I production transcripts and the potential to later develop bNAbs is very interesting. MHC-I presentation is usually seen as an immune pioneering process that then activates cytotoxic T cells (CTLs), thereby contributing to the early stages of the immune activation cascade. However, some viral proteins (e.g. HIV Nef https://www.nature.com/articles/nri2575) can downregulate MHC-I presentation as an escape mechanism from CTLs. These data suggests that perhaps in bNAb producers, early inhibition of MHC-I by viral factors is less effective, buying enough time for the immune system to take the bNAb route. If so, anti-Nef drugs or a Nef-mutant viral strain would be an interesting direction for vaccine efforts. The authors could test the hypothesis that patients with stronger MHC-I production early on (before returning to baseline) also have relatively fewer Nef reads (compared to the total of HIV reads) and/or mutations in Nef that might decrease its effectiveness (i.e. nonsynonymous mutations in relatively conserved residues of the protein). Potentially, the authors could also take the Nef sequences from samples with high vs low MHC-I production early on and predict structures using AlphaFold 3 (online server) to test the hypothesis that these specific Nef strains might differ structurally from the ones derived from non bNAbs producers.

We thank the reviewer for this thoughtful suggestion. We agree that the potential role of Nef-mediated modulation of MHC class I presentation in shaping early immune activation and bNAb development is highly relevant. In our dataset, however, we could not find evidence of differential read abundance in the *nef* region in relation to breadth, nor differences in the number of non-synonymous mutations within year of infection between bNAb producers and non-producers (Fig. R9). While this represents a promising direction, we believe it is best addressed in a dedicated follow-up study with targeted analyses and an expanded cohort.

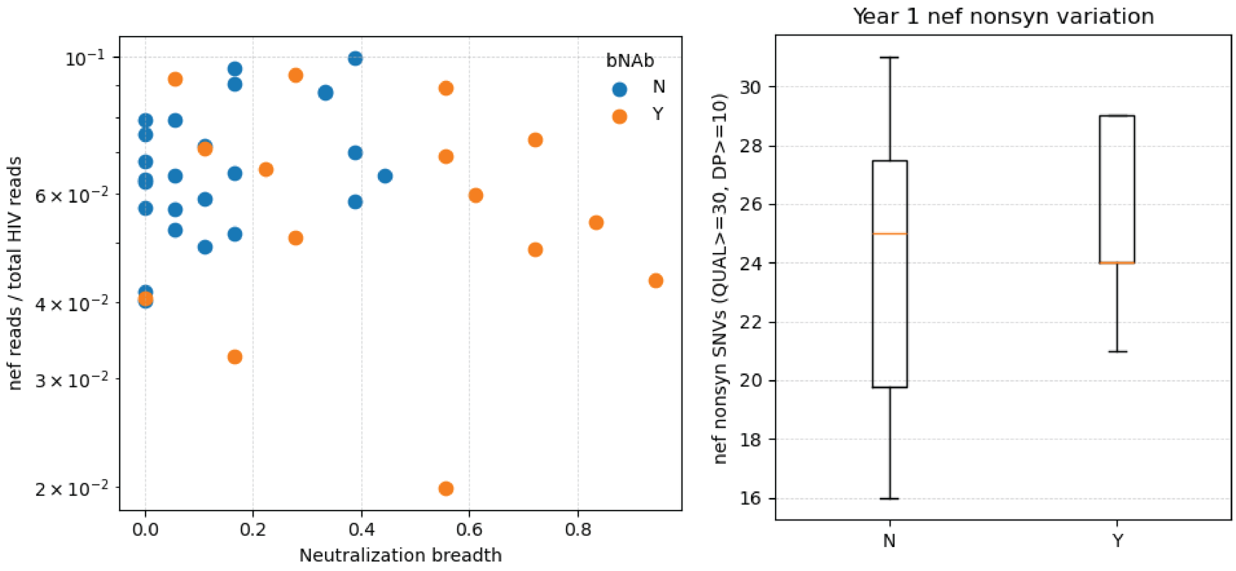

Figure R9. Analysis of Nef abundance and mutations. (Left) Proportion of reads mapping to the *nef* region relative to total HIV reads plotted against neutralization breadth. Samples are colored by bNAb status. (Right) Number of nonsynonymous single-nucleotide variants identified in the *nef* gene during the first year of infection in bNAb producers and non-producers.
